# Vacuum-Assisted Wound Therapy in Bothrops Snakebite Injuries: A Case Series from the Brazilian Amazon

**DOI:** 10.3390/jcm14228129

**Published:** 2025-11-17

**Authors:** Roberto C. C. Carbonell, Allan Q. Garcês-Filho, Luis E. B. Galan, Marcela Romanazzi, Geovanna M. Malachias-Pires, José R. Almeida, Jânio J. M. Nattrodt, Joquebede de L. B. Carbonell, Felipe A. Cerni, Sakthivel Vaiyapuri, Manuela B. Pucca

**Affiliations:** 1 Graduate Program in Bioscience and Biotechnology Applied to Pharmacy, School of Pharmaceutical Sciences, São Paulo State University (UNESP), Araraquara 19060-900, SP, Brazil; rcccarbonell@yahoo.es (R.C.C.C.); marcela15fernandes@gmail.com (M.R.); geovanna.malachias@unesp.br (G.M.M.-P.); 2 Medical School, Federal University of Roraima, Boa Vista 69310-000, RR, Brazil; allanquadros.ufrr@gmail.com (A.Q.G.-F.); luis.bermejo@ufrr.br (L.E.B.G.); felipe_cerni@hotmail.com (F.A.C.); 3 General Hospital of Roraima (HGR), Boa Vista 69305-455, RR, Brazil; janio_junior1@hotmail.com (J.J.M.N.); joquebedecarbonell@gmail.com (J.d.L.B.C.); 4 Biomolecules Discovery Group, Universidad Regional Amazónica Ikiam, Km 7 Via Muyuna, Tena 150101, Ecuador; j.r.dealmeida@reading.ac.uk; 5 School of Pharmacy, University of Reading, Reading RG6 6UB, UK; s.vaiyapuri@reading.ac.uk; 6 Department of Clinical Analysis, School of Pharmaceutical Sciences, São Paulo State University (UNESP), Araraquara 19060-900, SP, Brazil

**Keywords:** snakebite, *Bothrops* envenomation, vacuum-assisted wound therapy, wound healing, case series

## Abstract

In Brazil, snakebites are a critical health issue, particularly in the state of Roraima, where *Bothrops* species account for the majority of cases. Snake venoms contain multifunctional toxins that cause life-threatening systemic complications and complex wounds, often resulting in permanent disabilities. Although antivenoms have reduced lethality, they fail to prevent the rapid and prolonged action of tissue-damaging toxins at the bite site. As a result, snakebite management is characterized by prolonged hospitalizations and high healthcare costs. Therefore, effective management of these complex wounds is crucial to improving patient outcomes. This article presents a series of four clinical cases involving *Bothrops* snakebite patients treated at the General Hospital of Roraima. Each case describes the progression of the injury, clinical challenges, interventions, and outcomes. Vacuum-Assisted Wound Therapy (VAWT) was employed as a key component of treatment in all cases, promoting faster granulation tissue formation, infection control, and reduced risk of amputation. The use of VAWT demonstrated significant improvements in wound healing compared to conventional treatment approaches. These findings highlight the potential benefits of VAWT in managing complex snakebite-related wounds, especially in severe cases with high risks of complications. However, barriers such as the high cost of therapy and limited access in remote and resource-poor regions must be addressed to enable broader clinical application.

## 1. Introduction

Snakebite accidents pose a significant public health threat globally, classified by the World Health Organization (WHO) as a Category A Neglected Tropical Disease [1]. Each year, an estimated 5.4 million snakebite incidents occur worldwide, with around 2.7 million cases involving envenoming and leading to approximately 140,000 deaths annually [2]. Furthermore, individuals who suffer snakebites face a threefold increased risk of amputations and long-term debilitating sequelae, which can severely impact their quality of life [3]. The burden of snakebite injuries extends beyond the immediate health concerns, straining healthcare systems, particularly in low- and middle-income countries where access to medical care and antivenom is limited [4].

In Brazil, snakebite incidents represent a significant public health challenge, with over 180,000 reported cases between 2017 and 2022. The majority of these cases—approximately 80%—are attributed to the *Bothrops* genus (lancehead pit vipers), which are prevalent in the Amazon rainforest and surrounding areas. Other significant genera include *Crotalus* (rattlesnakes), which account for 8.5% of cases, *Lachesis* (bushmasters) and *Micrurus* (coral snakes), which contribute smaller percentages [5]. The state of Roraima, located in northern Brazil and bordering Venezuela and Guyana, records the highest incidence of snakebite accidents in the country, reflecting national trends that show a predominance of *B. atrox*. This species is endemic to the Amazon region and is responsible for the majority of venomous snakebites in northern Brazil and Roraima [6,7].

The clinical consequences of snakebite accidents are often severe, leading to serious complications such as necrosis, amputation, and acute kidney failure. Difficult-to-heal wounds are a common manifestation in snakebite victims, resulting from tissue destruction and disturbances in the regenerative niche caused by snake venom toxins and bacteria introduced by the bite [8,9]. These alterations hinder the successful healing process, resulting in substantial healthcare costs due to prolonged hospital stays, invasive procedures, and the need for costly medications, ultimately diminishing the quality of life for affected individuals [10]. In many cases, the delayed initiation of appropriate treatment can exacerbate these complications, potentially resulting in amputations and functional disabilities. Vacuum-assisted wound therapy (VAWT) has emerged as an effective strategy in managing complex wounds (e.g., snakebites), promoting healing and reducing the risk of amputation in severe cases.

Understanding the factors that predict poor prognosis in snakebite cases is essential for developing effective treatment protocols and mitigating long-term complications. This article reports on four cases of snakebite accidents that necessitated the use of vacuum-assisted wound therapy, examining the clinical challenges faced and the treatment outcomes achieved. By analyzing these cases, we aim to contribute valuable insights into snakebite management and the implications for clinical practice in regions where snakebites are prevalent, ultimately enhancing patient care and outcomes.

## 2. Case Series Presentation

### 2.1. CASE REPORT 1

**Figure 1:**
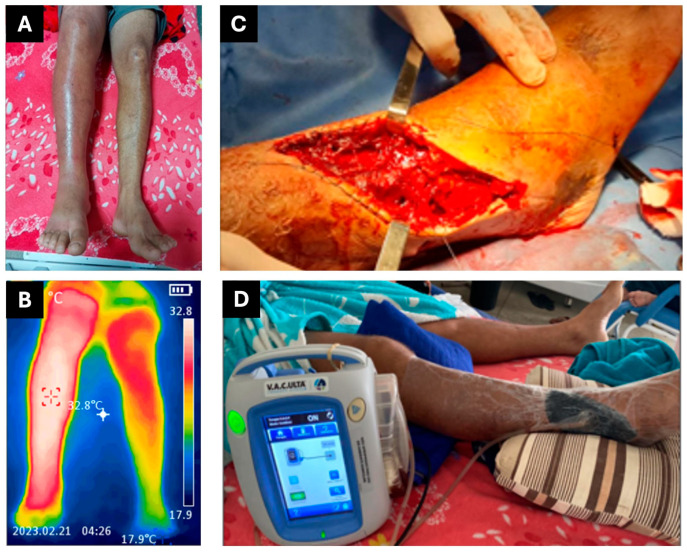
Manifestations of local snakebite damage and therapeutic interventions for patient 1. (**A**) Edema. (**B**) Thermography. (**C**) Fasciotomy and debridement. (**D**) VAWT.

On 16 February 2023 (day 1), a 38-year-old male patient from Brazil—a carpenter with no known comorbidities and a previously healthy medical history, married and father of four—was bitten on the right foot by a pit viper snake (*Bothrops* spp.), most likely *Bothrops atrox*, while fishing near Boa Vista, Roraima, as he was leaving the pasture around 7:00 p.m. He was admitted to the General Hospital of Roraima (HGR) at 10:30 p.m. on the same day and was administered six vials of antibothropic antivenom (SAB, *soro antibotrópico*, from Instituto Butantan, São Paulo, Brazil), classifying the envenoming as moderate. Upon admission, he presented with swelling extending to the knee, intense pain, no fever, preserved appetite, and normal urinary and bowel function. Upon arrival, laboratory tests revealed leukocytosis (26,500 white blood cells) and incoagulable blood (Table 1). On the second day after the bite (day 2), he was subsequently transferred to the Infectious Diseases Department of the General Hospital of Roraima (HGR). The affected limb measured 43 cm in circumference and had a temperature of 38 °C (Figure 1A), compared to 36 cm and 36.5 °C on the unaffected limb, with no signs of erythema or blisters. However, by day 6 (Figure 1B), the patient developed extensive erythema, blisters, and purulent exudate. On day 16, a CT scan of the affected area revealed an abscess with associated necrosis measuring approximately 19.4 × 6.3 × 3.7 cm, indicating the need for surgical drainage (performed on day 17) and initiation of antibiotic therapy with amikacin, teicoplanin, and ertapenem.

By day 20, the patient showed clinical and radiological signs of necrotizing fasciitis with associated muscle necrosis, prompting a recommendation for urgent surgical debridement. On day 21, a fasciotomy was performed to expose and manage the subcutaneous wound and to relieve elevated compartmental pressure in the affected limb (Figure 1C). VAWT at −125 mmHg was maintained from day 23 to day 29, with no dressing changes required during this period. After 40 days of treatment, including approximately one week with a negative pressure wound therapy system (Figure 1D), the patient was discharged on day 41 with a recommendation for outpatient follow-up care.

### 2.2. CASE REPORT 2

**Figure 2:**
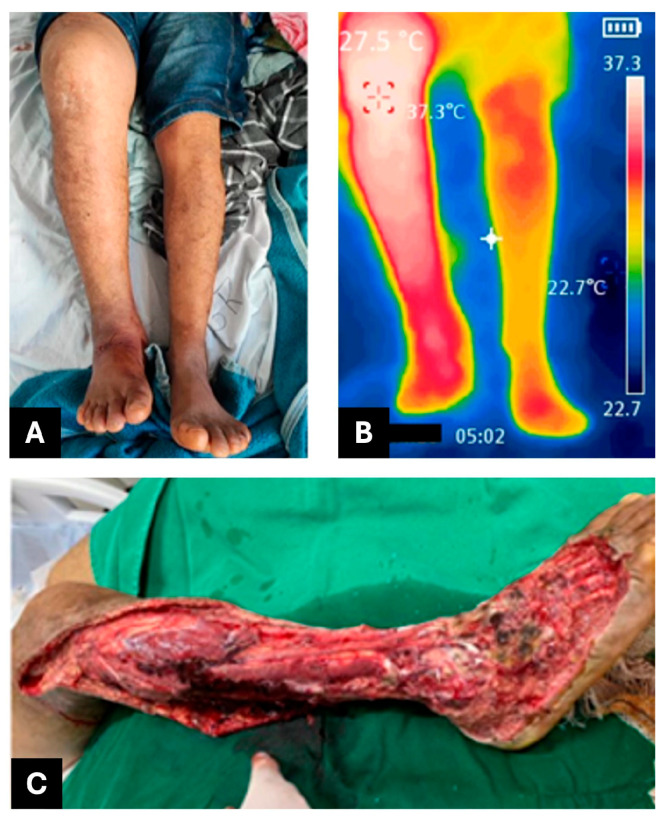
Manifestations of local snakebite damage and therapeutic interventions for patient 2. (**A**) Edema. (**B**) Thermography. (**C**) Fasciotomy and debridement.

This case describes a pit viper envenomation in a 28-year-old male patient. The *Bothrops atrox* bite occurred on 26 April 2023, near Boa Vista, Roraima, Brazil, at approximately 6:00 p.m., affecting the right lower limb (foot). The patient was admitted to HGR at 11:00 p.m. the same day, presenting with pain rated 10/10, extensive edema (40 cm in the affected limb compared to 36 cm in the contralateral limb), a mildly elevated temperature (37.3 °C compared to 36.9 °C) (Figure 2A), and gingival bleeding. Based on clinical findings, the envenomation was classified as severe. Following evaluation, the patient received 12 vials of antibothropic antivenom (SAB, *soro antibotrópico*, from Instituto Butantan), which were administered with a delay of about 5 h after the accident. He was subsequently transferred to the Infectious Diseases Department of the General Hospital of Roraima (HGR), where he was admitted on 27 April 2023 (day 1).

During the first five days of hospitalization in the Infectious Diseases Department, the patient continued to experience persistent severe pain, extensive ecchymosis, and febrile episodes, despite empirical treatment with amikacin and piperacillin–tazobactam, which proved clinically ineffective. Progressive edema and marked erythema were noted in the affected limb, indicating ongoing tissue inflammation and possible infection. Laboratory investigations revealed leukocytosis, elevated C-reactive protein (CRP), and evidence of coagulopathy, all suggestive of a systemic inflammatory response and evolving complications associated with envenomation (Table 2). Indeed, CRP levels indicated severe inflammation a few days post-incident, reaching 156.83 mg/L on April 30. Consequently, on 2 May 2023 (day 7), the patient was referred for fasciotomy and debridement (Figure 2C). During the procedure, he experienced hemodynamic instability (hypovolemic shock) and was admitted to the Intensive Care Unit (ICU), where he showed a good response to treatment and was discharged after three days.

**Figure 3:**
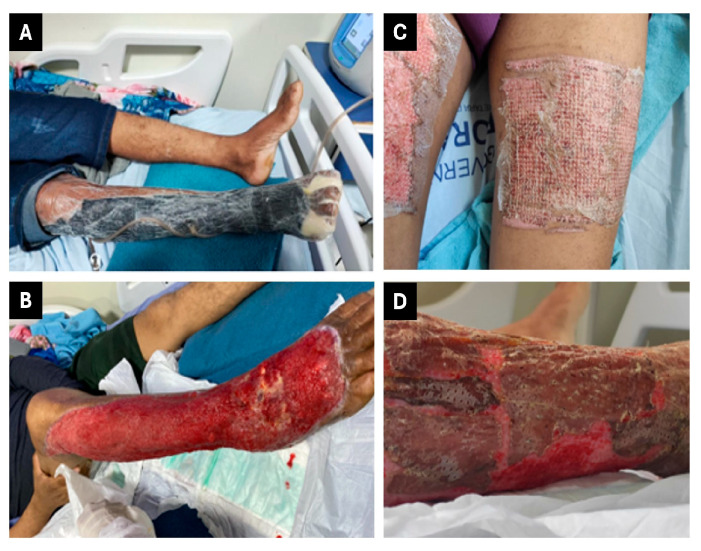
Therapeutic interventions for patient 2. (**A**) VAWT. (**B**) Wound appearance following vacuum dressing treatment, demonstrating improved tissue viability. (**C**) Harvest site of the split-thickness skin graft (donor area). (**D**) Placement of the skin graft over the debrided wound bed to promote definitive wound closure.

On 8 May 2023 (day 13), VAWT at −125 mmHg was initiated (Figure 3A). The patient continued with the vacuum dressing until 23 May 2023 (15 days of use), demonstrating rapid granulation tissue formation and satisfactory progress (Figure 3B). Subsequently, the patient began daily dressings with collagenase every 12 h. On 12 June 2023 (day 44), he underwent surgery for graft placement at the wound site (Figure 3C,D). The patient progressed well, requiring only simple dressings after the grafting procedure performed by the plastic surgery team. On 26 June 2023 (60 days post-accident), he was discharged with instructions for outpatient follow-up and wound care.

### 2.3. CASE REPORT 3

A 61-year-old male patient with a history of systemic hypertension and insulin-dependent type 2 diabetes mellitus was bitten by a *Bothrops atrox* on 24 May 2023, in Uiramutã, a rural municipality in the state of Roraima, Brazil. He was referred to HGR for envenoming management (day 1). On admission, he presented with an extensive wound on his left foot, accompanied by swelling, redness, blood-filled blisters, and severe pain. The envenomation was classified as severe, warranting the administration of 12 vials of antibothropic antivenom (SAB, *soro antibotrópico*, from Instituto Butantan). On day 2, the patient was subsequently transferred to the Infectious Diseases Department of the General Hospital of Roraima (HGR), admitted for inpatient clinical care and close monitoring due to the risk of systemic complications such as coagulopathy, secondary infection, and tissue necrosis. Laboratory tests at the time of hospitalization showed marked leukocytosis with a left shift, elevated C-reactive protein (CRP), and worsening renal function, but no abnormalities in the coagulation profile (Table 3).

**Figure 4:**
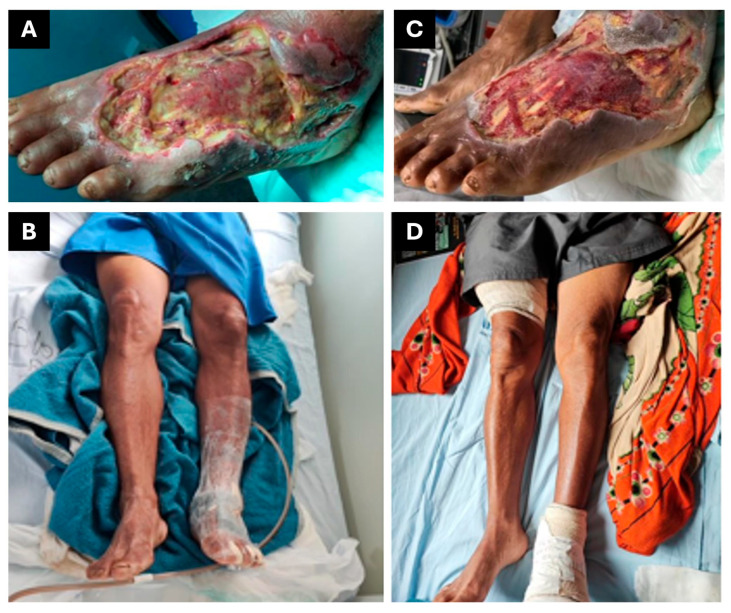
Therapeutic interventions for patient 3. (**A**) Fasciotomy and debridement. (**B**) VAWT. (**C**) Wound appearance following vacuum dressing treatment, demonstrating improved tissue viability. (**D**) Postoperative recovery with compression bandages applied to both donor and recipient sites following the skin graft.

On day 2, the patient was started on intravenous ampicillin–sulbactam due to clinical signs of wound necrosis and secondary bacterial infection. On 30 May 2023 (day 7), the plastic surgery team performed surgical debridement to remove devitalized tissue and recommended the use of topical collagenase-based enzymatic dressings for ongoing wound management. By 3 June 2023 (day 11), laboratory investigations indicated partial control of the infection, as reflected in the reduction in inflammatory markers (Table 3). On the same day, VAWT at −125 mmHg was initiated (Figure 4A) and maintained until 20 June 2023 (day 29), in association with continued antibiotic treatment. During this period, the patient remained under evaluation by the plastic surgery team in preparation for definitive closure with a skin graft (Figure 4B–D).

On 17 July 2023 (day 56), a skin graft was successfully performed on the wound, which had fully healed from the infection, using donor tissue from the right thigh. Following the procedure, the patient was discharged home with instructions for wound care and scheduled outpatient follow-up. It is important to highlight that the delay in this case was not due to complications with wound healing, but rather a delay in the scheduling of the graft procedure by the plastic surgery team.

### 2.4. CASE REPORT 4

**Figure 5:**
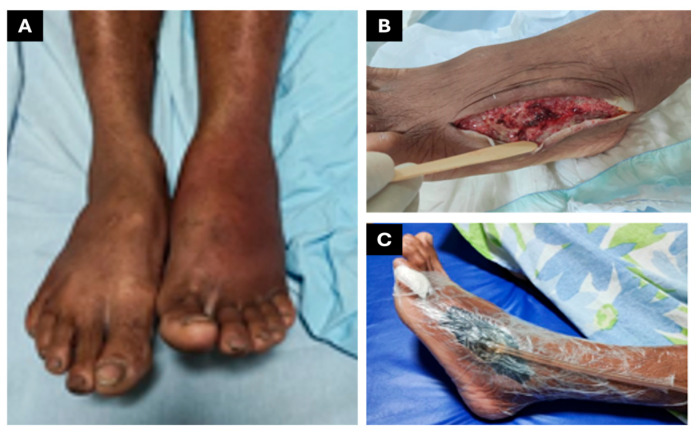
Manifestations of local snakebite damage and therapeutic interventions for patient 4. (**A**) Edema. (**B**) Fasciotomy and debridement. (**C**) VAWT.

A 36-year-old male patient, originally from Boa Vista and currently residing in Rorainópolis, Roraima, Brazil, with a known history of asthma but no other significant comorbidities, was bitten by a snake on 11 May 2023 (day 1). The patient was unable to identify the species responsible for the envenomation. On the same day, the patient was admitted to HGR for envenomation management. Clinical examination revealed edema, erythema, and increased warmth in the left lower limb (Figure 5A,B), accompanied by fever but without signs of neurotoxicity. These findings excluded rattlesnake-induced envenomation and supported the classification of the case as a moderate *Bothrops* envenomation. The patient was treated with six vials of antibothropic antivenom (SAB, *soro antibotrópico*, from Instituto Butantan). The patient was subsequently transferred to the Infectious Diseases Department of the General Hospital of Roraima (HGR).

During hospitalization, antibiotic therapy was initiated due to clinical and laboratory signs of infection (Table 4), with Amoxicillin + Clavulanate started on day 1 and Amikacin introduced on day 3. On day 5, an ultrasound was performed, which revealed free fluid in a laminar pattern in the leg and dorsum of the foot, with coarse infiltration present in greater quantities than in the other areas examined. On day 6, a consultation was requested with the hospital’s wound care team to assess the need for surgical intervention, including debridement and fasciotomy to relieve internal pressure (Figure 5C). On Day 8, the procedure was successfully carried out without complications, and a collagenase dressing was temporarily prescribed.

On day 13 of hospitalization, vacuum-assisted wound therapy at −125 mmHg was recommended to facilitate the healing of the lesion, and it was maintained until day 16. Given the positive progress of the wound and the improvement in the infection, the patient was discharged home with instructions for wound care and a follow-up appointment for reevaluation scheduled one month later.

## 3. Discussion

VAWT has emerged as an innovative and effective strategy for managing complex wounds resulting from snakebite envenomation, particularly those caused by *Bothrops* species, which are highly prevalent in Brazil [11]. These accidents pose a significant public health challenge due to the severity of local tissue complications, including necrosis, secondary infections, and vascular impairment [12]. The venom of *Bothrops* snakes induces tissue destruction through multiple mechanisms (e.g., cytotoxicity, hemorrhagic activity, degradation of the extracellular matrix, and procoagulant effects), which often lead to progressive damage, especially when the administration of antivenom is delayed [13,14].

Conventional snakebite wound management typically involves standard dressings and surgical procedures. However, these approaches often show limited efficacy in infection control and wound resolution [15]. In contrast, VAWT has demonstrated benefits in accelerating the healing process, promoting granulation tissue formation, and reducing the risk of amputation. This is particularly relevant in patients with comorbidities such as diabetes mellitus and hypertension, who are more vulnerable to complications [16,17]. Nonetheless, VAWT implementation is limited by its high cost and the need for specialized infrastructure, which can be challenging in remote or resource-limited settings [18].

To date, no studies have specifically documented the use of VAWT in snakebite-related wounds [19]. However, negative pressure wound therapy (NPWT)—the broader category under which VAWT falls—has been extensively studied and applied in various types of wounds, demonstrating significant effectiveness in promoting healing. NPWT has shown favorable results in the management of chronic ulcers, traumatic injuries, and postoperative wounds [20]. In cases of open fractures, NPWT has been effective in exudate removal, infection control, and stimulation of granulation tissue, thus facilitating wound closure even in the presence of extensive soft tissue damage [21].

NPWT has also proven beneficial in improving skin graft outcomes by enhancing adherence to the wound bed, increasing vascularization, and reducing the risk of graft detachment. These effects are crucial in reconstructive procedures and the treatment of burns or chronic wounds requiring skin coverage [22]. Additional applications include diabetic ulcers, where NPWT promotes granulation and reduces wound size [23]; pressure ulcers, by improving local perfusion and reducing infections; and burns, by supporting tissue regeneration in a moist environment. NPWT also aids in managing infected wounds through bacterial load control and wound cleansing [24]. Furthermore, NPWT has been applied to surgical wound dehiscence and open abdomen management, helping to prevent severe complications by optimizing the wound environment [25,26].

In the present case series, VAWT was successfully used in four patients with severe *Bothrops* envenomation and complex wounds. All patients initially presented with classic local signs of envenomation—edema, erythema, localized warmth, and hemorrhagic blisters—and received prompt administration of anti-*Bothrops* antivenom (SAB, Instituto Butantan). Notably, significant improvement in inflammatory parameters was observed only after the initiation of VAWT. This aligns with previous studies that support the efficacy of NPWT in complex wounds, where it promotes granulation tissue formation and facilitates surgical wound closure [27,28,29].

Among the analyzed patients, those with underlying comorbidities—particularly diabetes mellitus—faced greater challenges in the healing process. The case of a 61-year-old patient with type 2 diabetes exemplified this, presenting with prolonged inflammation, persistent elevation of C-reactive protein (CRP), and delayed normalization of hematological parameters. The patient also experienced difficulty in controlling local infection and necrosis. These findings are consistent with the literature, which highlights impaired wound healing in diabetic patients due to microvascular dysfunction, peripheral neuropathy, and reduced immune response [30,31]. Despite these obstacles, the initiation of VAWT led to a marked reduction in CRP levels, leukocyte counts, and neutrophils, suggesting effective control of the inflammatory process and improved wound status. These results support prior studies showing that VAWT enhances tissue vascularization and reduces infection risk in diabetic patients, thereby promoting faster healing compared to conventional therapies [32].

Comparing outcomes among patients with and without significant comorbidities reveals that overall health status influences response to treatment. In patients without chronic conditions—such as the 34-year-old case—there was a faster reduction in inflammatory markers following VAWT, and a more rapid clinical improvement. This suggests that baseline physiological resilience may enhance the benefits of the therapy, even though VAWT remains effective across different patient profiles.

Beyond physical recovery, VAWT also significantly impacts patients’ quality of life. Chronic or complex wounds are often associated with mobility limitations, persistent pain, social isolation, and the risk of severe complications such as sepsis [33]. By accelerating the healing process, VAWT helps reduce these burdens, enabling patients to return to daily activities and improving emotional well-being [34]. In diabetic patients, it has even been shown to prevent amputations—an outcome with major psychological and functional consequences [35]. Furthermore, patients without comorbidities, such as the 28-year-old in this series, also reported significant improvements in mobility and pain following VAWT, reinforcing its broader applicability.

Although VAWT use in snakebite-related wounds is still underexplored in the literature, the cases presented here highlight its potential as a valuable therapeutic tool. Most prior studies on VAWT have focused on chronic wounds of different etiologies, such as pressure ulcers and diabetic foot ulcers, which are similarly complicated by persistent inflammation and secondary infections [36,37]. These studies have also emphasized the economic advantages of VAWT, including shorter hospital stays and fewer surgical interventions, which ultimately reduce healthcare costs.

## 4. Conclusions

Based on the results obtained in this study, potential future applications for VAWT can be suggested, including the management of snakebite wounds in remote and resource-limited areas, provided there is adequate investment in infrastructure and professional training. Furthermore, controlled clinical studies could more comprehensively assess the cost-effectiveness relationship of this technique in different contexts, such as wounds caused by other venomous animals or in patients with multiple comorbidities.

In conclusion, VAWT has proven to be a powerful tool in managing complex wounds, standing out for its ability to promote efficient healing even under challenging clinical conditions, such as diabetes mellitus. Although there are still barriers to its widespread implementation, the clinical benefits and positive impact on patients’ quality of life reinforce the need to expand its use, particularly in regions where snakebites are frequent.

## Figures and Tables

**Table 1:** Laboratory investigation reports for snakebite patient 1 during hospitalization. Altered levels are highlighted in bold.

Analytes *	16/02 00:07	16/02 09:46	16/02 20:03	26/02	01/03	03/03 Before VAWT Therapy	09/03 During VAWT Therapy	Reference Range **
Hemoglobin ^1^	15.6	15.9	15	**12.3**	**11.3**	**10.8**	**9.4**	13.5–18.0 g/dL
Leucocytes ^1^	**26,500**	**21,400**	**21,500**	**18,650**	**17,590**	**16,360**	**11,200**	4000–10,000 cells/µL
Neutrophils ^1^	**91%**	**82%**	**79%**	**76.3%**	**76.3%**	**74.2%**	64.2%	50–70%
Platelets ^1,^***	283,000	303,000	239,000	**684,000**	**776,000**	**748,000**	**500,000**	150,000–400,000/µL
PT ^2^	**Uncoagulable**	-	**14.5**	13.2	13.7	13.0	-	10–14 s
aPTT	**48.9**	-	31.8	25.4	29.3	29.6	-	25–39 s
Urea ^3^	31	30	30	23.64	27.79	27.09	17.63	16–40 mg/dL
Creatinine ^4^	1.0	0.7	1.0	0.84	0.97	0.85	0.94	0.7–1.4 mg/dL
ALT ^3^	-	-	**54**	**51.73**	42.35	30.11	17.95	5–48 U/mL
AST ^5^	-	-	**87**	34.42	31.31	24.08	15.54	5–48 U/mL
CRP ^6^	-	-	-	**78.67**	**66.96**	**98.5**	**14.82**	0.0–8.0 mg/L

* Laboratory tests collected and analyzed by the *Laboratório Central de Roraima* (LACEM-HGR). ** Reference range from the *Laboratório Central de Roraima* (LACEM-HGR), Boa Vista, Roraima, Brazil. *** The platelet results are rounded by the laboratory to ensure there are no decimal places. ^1^ Automated analysis (SF CUBE/BC-6200™); ^2^ Automated analysis (Destiny Plus™); ^3^ Kinetic-UV METHOD; ^4^ Kinetic Alkaline Picrate Method; ^5^ Enzymatic-Colorimetric (Trinder); ^6^ Immunoturbidimetry. ALT: alanine aminotransferase; AST: aspartate aminotransferase; CRP: C-reactive protein; PT: prothrombin time; INR: international normalized ratio.

**Table 2:** Laboratory investigation reports for snakebite patient 2 during hospitalization. Altered levels are highlighted in bold.

Analytes *	27/04	30/04	06/05	13/05	21/05	28/05	16/06	23/06	Reference Range **
Hemoglobin ^1^	14.4	11.3	**6.5**	**9.5**	**9.6**	**9.6**	**8.6**	**9.8**	13.5–18.0 g/dL
Leucocytes ^1^	**22,900**	**16,980**	**18,610**	**11,460**	8140	4870	9200	5970	4000–10,000 cells/µL
Neutrophils ^1^	**86.5%**	**81.9%**	**76.6%**	69.4%	60%	**25%**	**62.2%**	**41.8%**	50–70%
Platelets ^1,^***	**383,000**	**89,000**	**785,000**	**1019,000**	**599,000**	**571,000**	**449,000**	**479,000**	150,000–400,000/µL
PT ^2^	-	**23.9**	12.4	12.1	13.0	12.9	-	-	10–14 s
aPTT	-	**47.1**	28.7	30.0	31.7	36.3	-	-	25–39 s
Urea ^3^	32.56	20.81	19.51	17.45	18.53	**13.8**	17	24	16–40 mg/dL
Creatinine ^4^	0.8	0.96	0.78	0.81	0.71	0.87	0.8	0.8	0.7–1.4 mg/dL
ALT ^3^	37.68	25.94	-	**76.81**	45.49	42.8	39	**54**	5–48 U/mL
AST ^5^	39.42	46.66	-	32.85	19.76	22.69	18	30	5–48 U/mL
CRP ^6^	2.67	**156.83**	**79.31**	2.77	3.88	**67**	1.7	0.75	0.0–8.0 mg/L

* Laboratory tests collected and analyzed by the *Laboratório Central de Roraima* (LACEM-HGR). ** Reference range from the *Laboratório Central de Roraima* (LACEM-HGR), Boa Vista, Roraima, Brazil. *** The platelet results are rounded by the laboratory to ensure there are no decimal places. ^1^ Automated analysis (SF CUBE/BC-6200™); ^2^ Automated analysis (Destiny Plus™); ^3^ Kinetic-UV METHOD; ^4^ Kinetic Alkaline Picrate Method; ^5^ Enzymatic-Colorimetric (Trinder); ^6^ Immunoturbidimetry. ALT: alanine aminotransferase; AST: aspartate aminotransferase; CRP: C-reactive protein; PT: prothrombin time; INR: international normalized ratio.

**Table 3:** Laboratory investigation reports for snakebite patient 3 during hospitalization. Altered levels are highlighted in bold.

Analytes *	24/05	27/05	03/06	09/06	16/06	23/06	11/07	17/07	Reference Range **
Hemoglobin ^1^	14.9	**12.4**	**11**	**10.6**	**10.9**	**10.9**	**11.8**	**11.0**	13.5–18.0 g/dL
Leucocytes ^1^	**23,980**	**12,290**	5380	6230	4620	**3200**	5200	5270	4000–10,000 cells/µL
Neutrophils ^1^	**94%**	**81%**	69.2%	68%	52.6%	**41.5%**	**49%**	57.3%	50–70%
Platelets ^1,^***	192,000	227,000	**418,000**	**493,000**	309,000	216,000	284,000	208,000	150,000–400,000/µL
PT ^2^	**14.2**	**15.3**	**14.3**	13.7	14	-	-	-	10–14 s
aPTT	35.4	32.6	32.5	33.3	32.9	-	-	-	25–39 s
Urea ^3^	**91.22**	-	26.9	26	17	**9.4**	**14**	21.63	16–40 mg/dL
Creatinine ^4^	**1.98**	0.74	0.77	0.92	0.97	0.67	0.7	**0.59**	0.7–1.4 mg/dL
ALT ^3^	33.18	-	41.57	33.2	24	29	14	14.9	5–48 U/mL
AST ^5^	37	15.65	32.47	33.1	22	25	15	11.89	5–48 U/mL
CRP ^6^	**165**	**139**	**58.4**	**50**	**9.69**	4	1.52	1.06	0.0–8.0 mg/L

* Laboratory tests collected and analyzed by the *Laboratório Central de Roraima* (LACEM-HGR). ** Reference range from the *Laboratório Central de Roraima* (LACEM-HGR), Boa Vista, Roraima, Brazil. *** The platelet results are rounded by the laboratory to ensure there are no decimal places. ^1^ Automated analysis (SF CUBE/BC-6200™); ^2^ Automated analysis (Destiny Plus™); ^3^ Kinetic-UV METHOD; ^4^ Kinetic Alkaline Picrate Method; ^5^ Enzymatic-Colorimetric (Trinder); ^6^ Immunoturbidimetry. ALT: alanine aminotransferase; AST: aspartate aminotransferase; CRP: C-reactive protein; PT: prothrombin time; INR: international normalized ratio.

**Table 4:** Laboratory investigation reports for snakebite patient 4 during hospitalization. Altered levels are highlighted in bold.

Analytes *	11/05	12/05	14/05	20/05	Reference Range **
Hemoglobin ^1^	15.3	16.1	15.7	15.7	13.5–18.0 g/dL
Leucocytes ^1^	9410	**14,740**	**14,960**	7890	4000–10,000 cells/µL
Neutrophils ^1^	67.5%	**76.8%**	**80.3%**	58.3%	50–70%
Platelets ^1,^***	192,000	227,000	**418,000**	**493,000**	150,000–400,000/µL
PT ^2^	**14.9**	13.8	12.8	**14.2**	10–14 s
aPTT	31.2	27.6	29.8	30.7	25–39 s
Urea ^3^	33.66	26.07	16.91	35.18	16–40 mg/dL
Creatinine ^4^	0.87	1.19	1.01	1.14	0.7–1.4 mg/dL
ALT ^3^	27.62	34.09	37.62	38.54	5–48 U/mL
AST ^5^	29.51	**58.95**	30.44	23.41	5–48 U/mL
CRP ^6^	0.47	**59.27**	**157.09**	**25.79**	0.0–8.0 mg/L

* Laboratory tests collected and analyzed by the *Laboratório Central de Roraima* (LACEM-HGR). ** Reference range from the *Laboratório Central de Roraima* (LACEM-HGR), Boa Vista, Roraima, Brazil. *** The platelet results are rounded by the laboratory to ensure there are no decimal places. ^1^ Automated analysis (SF CUBE/BC-6200™); ^2^ Automated analysis (Destiny Plus™); ^3^ Kinetic-UV METHOD; ^4^ Kinetic Alkaline Picrate Method; ^5^ Enzymatic-Colorimetric (Trinder); ^6^ Immunoturbidimetry. ALT: alanine aminotransferase; AST: aspartate aminotransferase; CRP: C-reactive protein; PT: prothrombin time; INR: international normalized ratio.

## Data Availability

Data available on request from the authors.
